# Unusual coexistence: branchial cleft cyst harboring papillary thyroid carcinoma with lymph node metastasis — a rare case report and clinical insights

**DOI:** 10.3389/fonc.2024.1378405

**Published:** 2024-04-11

**Authors:** Wei-Tao Wang, Xi-Hao Ni, Yong-Xue Gu, Ran An, Chang-Liang Wang, Jun Zhang

**Affiliations:** ^1^ School of Clinical Medicine, Shandong Second Medical University, Weifang, Shandong, China; ^2^ Department of Thyroid and Breast Surgery, Weifang People’s Hospital, Weifang, Shandong, China

**Keywords:** papillary thyroid carcinoma, branchial cleft cyst, lymph node metastasis, case report, clinical insights

## Abstract

**Background:**

The simultaneous occurrence of Branchial Cleft Cyst (BCC) and Papillary Thyroid Carcinoma (PTC) represents an unusual malignant tumor, with cases featuring associated lymph node metastasis being particularly rare. This combination underscores an increased potential for metastasis, and the assessment of neck masses, particularly on the lateral aspect, may inadvertently overlook the scrutiny of the thyroid. Therefore, healthcare providers should exercise vigilance, especially in patients over the age of 40, regarding the potential for neck masses to signify metastasis from thyroid malignancies. Currently, surgical intervention stands as the primary effective curative method, while the postoperative administration of radioactive iodine therapy remains a topic of ongoing debate.

**Case report:**

In the presented case, a 48-year-old male patient with a right neck mass underwent surgical intervention. The procedure included the excision of the right neck mass, unilateral thyroidectomy with isthmus resection, and functional neck lymph node dissection under tracheal intubation and general anesthesia. Postoperative pathology findings revealed the coexistence of a BCC with metastatic PTC in the right neck mass, as well as papillary carcinoma in the right thyroid lobe. Lymph node metastasis was observed in the central and levels III of the right neck.

**Conclusion:**

The rare amalgamation of a BCC with PTC and concurrent lymph node metastasis underscores the invasive nature of this malignancy. Healthcare professionals should be well-acquainted with its clinical presentation, pathological characteristics, and diagnostic criteria. A multidisciplinary approach is strongly recommended to enhance patient outcomes.

## Introduction

BCC are the second most common congenital neck lesions following thyroglossal duct cysts (1). BCC and their fistulas result from abnormal development of branchial grooves or pouches during embryonic development. In the human embryo, there are four pairs of distinct branchial grooves and pouches, with the first groove forming the external auditory canal, and the second, third, and fourth grooves gradually merging and disappearing. If the fusion process of any of the first to fourth branchial grooves is abnormal, leading to incomplete closure, it can give rise to corresponding branchial cysts and fistulas. Most BCC originate from the second branchial arch, typically located in the upper third of the anterior triangle of the neck, specifically in the sternocleidomastoid muscle region, and rarely occur in other locations (2).

PTC stands as the predominant histological variant among thyroid cancers, constituting 70-80% of all cases. It commonly manifests in individuals during their third and fourth decades, with a prevalence that is twice as high in females compared to males. Occult cancers are typically smaller than 1.5 cm, not palpable, and frequently discovered incidentally. The majority of patients exhibit a painless, gradually expanding mass. This cancer variant tends to spread to the peritracheal and cervical lymphatics (3). Papillary carcinoma has a tendency for lymphatic metastasis, and metastatic lymph nodes are often detectable even when the primary tumor is not clearly apparent (4). Although uncommon, cystic lymph node metastasis in the neck can occur, and PTC may manifest as lateral neck cysts resembling BCC (5).

To enhance awareness among clinical professionals, we present the clinical data of a patient with concurrent BCC and PTC with lymph node metastasis, admitted to the Thyroid and Breast Surgery Department of Weifang People’s Hospital. The details are reported below.

## Case report

**Figure 1:**
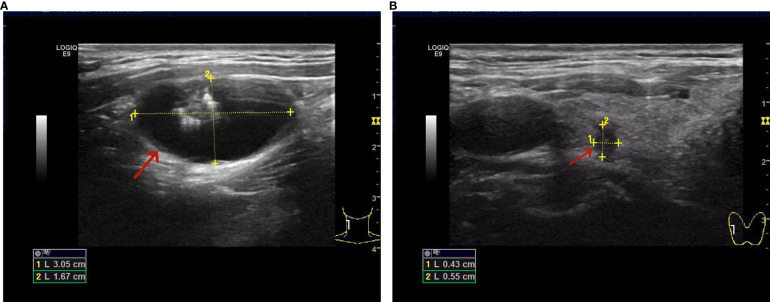
**(A)** Solid-cystic nodule with calcification in the right side of the neck. **(B)** There is a solid nodule in the upper pole of the right lobe of the thyroid, measuring approximately 0.4*0.6 cm.

**Figure 2:**
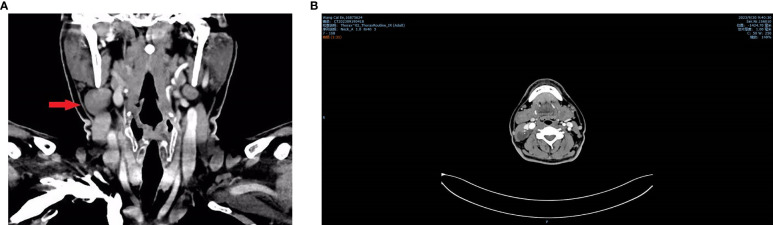
Neck CT plain scan + enhancement shows a solid-cystic mass with calcification in the right side of the neck. **(A)** Coronal view. **(B)** Horizontal view.

**Figure 3:**
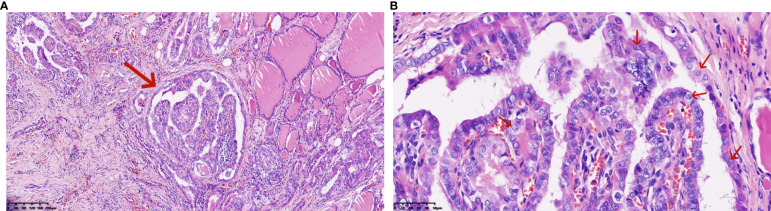
**(A, B)** Pathological features of right thyroid lobe carcinoma (Hematoxylin and eosin stain): The papillary carcinoma consists of tumor cells with eosinophilic cytoplasm, demonstrating typical ground-glass nuclei, overlapping nuclei, nuclear grooves, and intranuclear inclusions (*100, *400).

**Figure 4:**
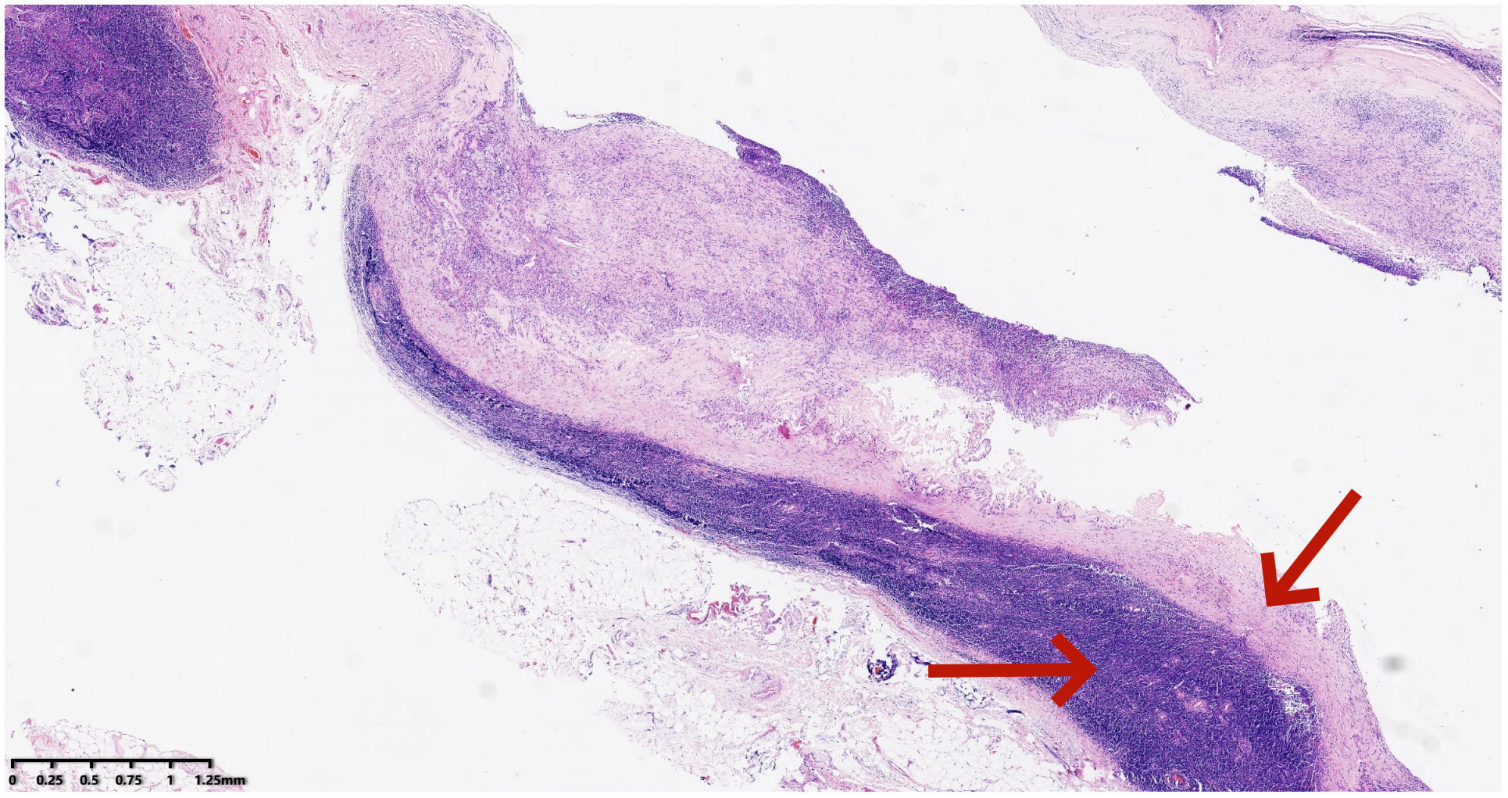
Pathological features of the right neck mass showing branchial cleft cyst (Hematoxylin and eosin stain): Lined by a single layer of epithelium, with lymphoid tissue located beneath the epithelium (*20).

**Figure 5:**
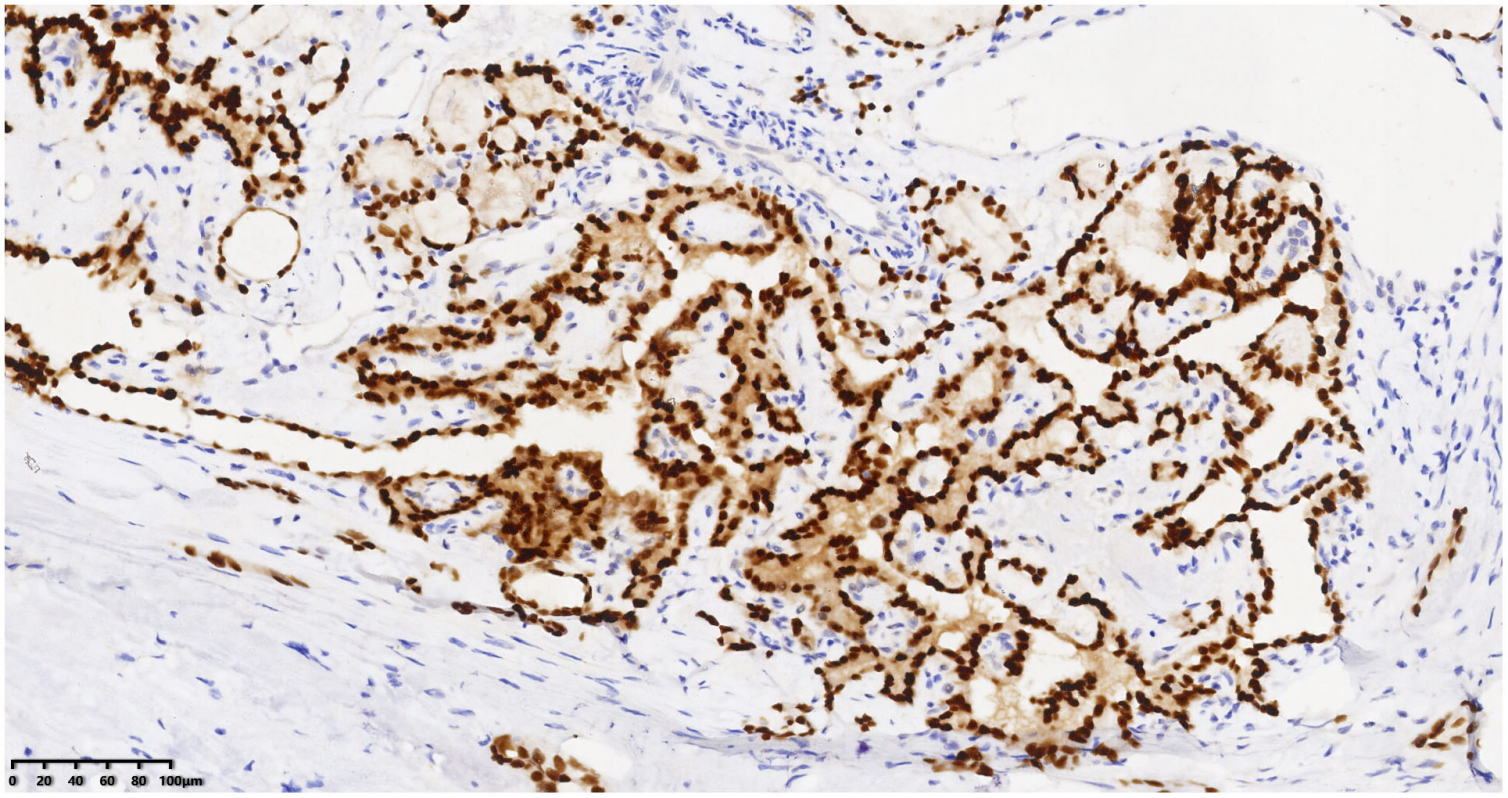
Right neck mass with positive PAX-8 staining, immunohistochemical envision (*200).

**Figure 6:**
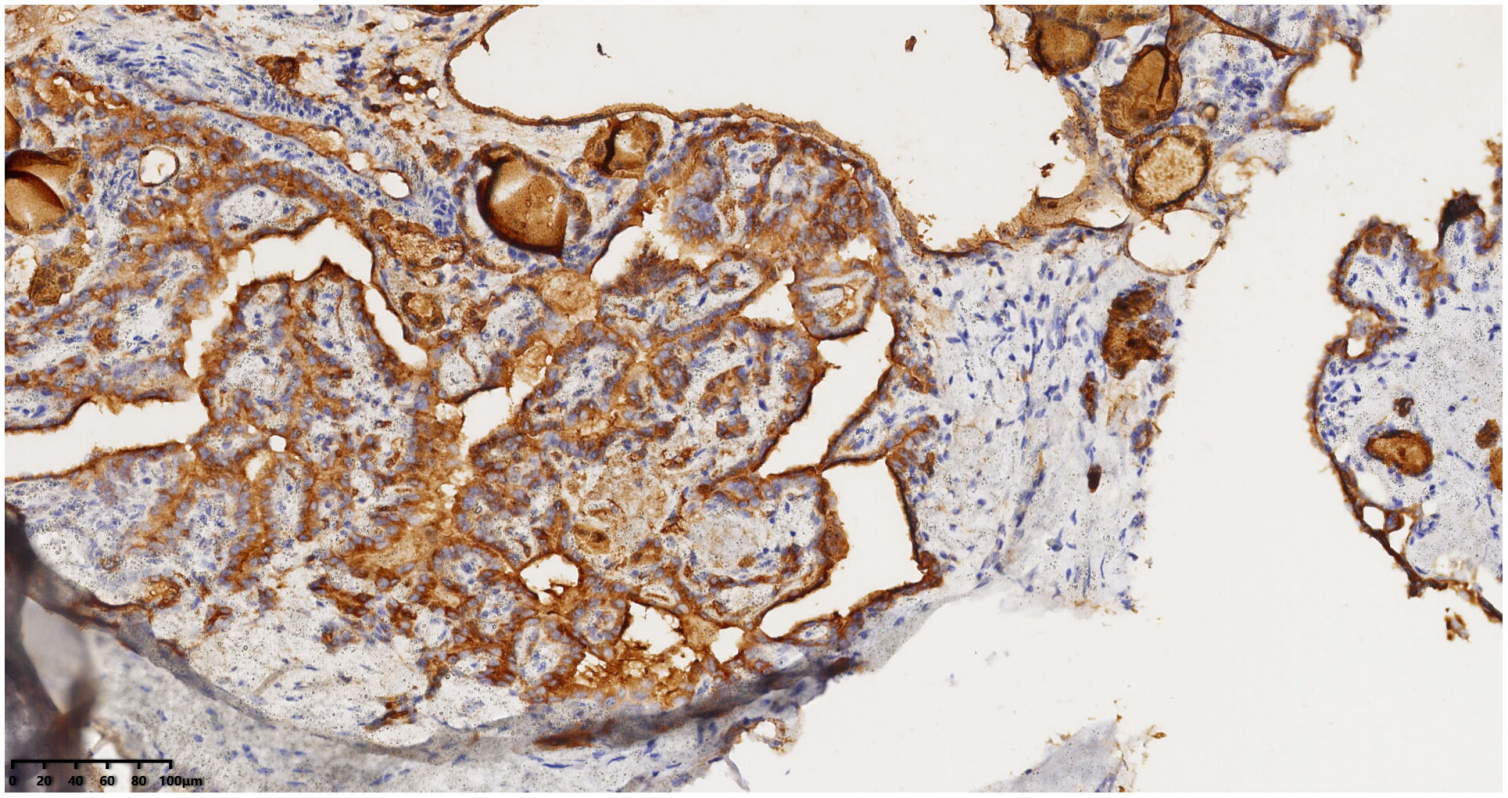
Right neck mass with positive TG staining, immunohistochemical envision (*200).

**Figure 7:**
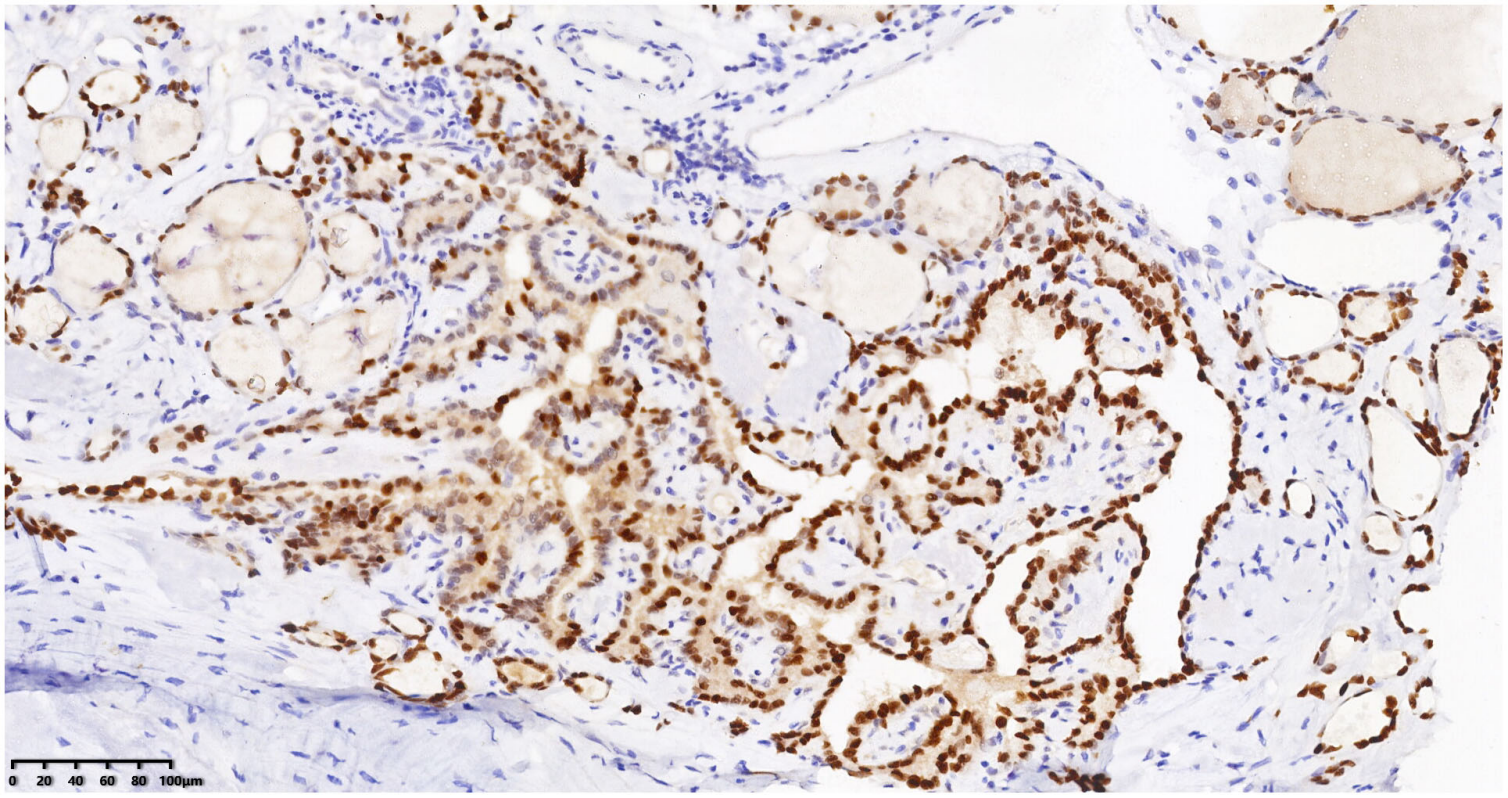
Right neck mass with positive TTF-1 staining, immunohistochemical envision (*200).

**Figure 8:**
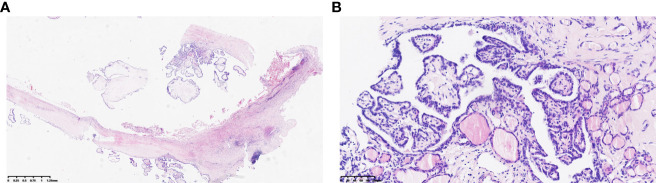
**(A, B)** Branchial cleft cyst with concurrent metastasis of papillary thyroid carcinoma (Hematoxylin and eosin stain) (*20, *200).

The patient, a 48-year-old male, presented to the Thyroid and Breast Surgery Department of Weifang People’s Hospital on September 8, 2023, complaining of a right neck mass discovered six months prior, which he perceived to be progressively enlarging. Clinical examination revealed a palpable, firm, well-defined, smooth-surfaced mass measuring approximately 3*3 cm behind the sternocleidomastoid muscle, with no tenderness or local skin erythema. Thyroid ultrasound indicated a solid nodule in the right thyroid lobe categorized as ACR TI-RADS 5, a solid cystic nodule in the right thyroid lobe categorized as ACR TI-RADS 2, and a cystic solid nodule with calcification in the right neck (**Figures 1A, B**). A CT scan confirmed a solid-cystic lesion with calcification in the right neck, suggestive of a benign lesion, possibly of vascular origin, along with multiple small lymph nodes in bilateral neck and subclavicular regions (**Figures 2A, B**). Considering these findings, the patient underwent general anesthesia and tracheal intubation for the removal of the right neck mass, unilateral thyroidectomy with isthmus resection, and functional neck lymph node dissection. Postoperative pathology revealed PTC in the right thyroid lobe and isthmus, accompanied by fibrosis, with a diameter of 0.3 cm and invasion of the capsule. No definite evidence of neural invasion or intravascular tumor thrombus was observed. Lymphocytic thyroiditis was also identified. Examination of the excised lymph nodes showed metastasis (0/3 in pretracheal, 1/1 in right central, 0/2 in right level II, 1/10 in right level III, and 0/7 in right level IV). Additionally, a small amount of thyroid and skeletal muscle tissue with cancer components was found in the pretracheal specimen, while the right level II lymph node specimen contained a small amount of salivary gland tissue without cancer components. In the right neck, a cystic lesion measuring 3*2*1.8 cm was confirmed to be a BCC. Notably, calcifications and papillary-like structures were identified on the inner wall of the BCC. Pathology and immunohistochemical results revealed positive expression of TG, TTF-1, and PAX-8 in the BCC, indicating metastasis of PTC (**Figures 3A, B**, **4**–**8**). The patient’s postoperative recovery has been satisfactory, and regular follow-up examinations are currently ongoing.

## Discussion

BCC are relatively common neck masses clinically, occurring at any age, with a higher prevalence around the age of 30, and affecting both males and females. However, the discovery of PTC within a BCC is rare. The simultaneous occurrence of PTC within a BCC with associated lymph node metastasis is exceptionally rare (1). Based on a retrospective analysis of previous cases, neck masses related to PTC can be categorized into the following four types: cystic lymph node metastasis of PTC, ectopic PTC within a BCC, BCC with concurrent PTC, and BCC with concurrent PTC and lymph node metastasis.

Differential diagnoses for lateral neck masses include inflammatory conditions such as cat scratch disease, infectious mononucleosis, sialadenitis, or other reactive lymphadenopathies; congenital anomalies such as cystic hygroma or BCC, fistulas, and abscesses; and neoplastic conditions such as lymphoma, lymphatic malformations, neurofibromas, salivary gland tumors, and various metastatic lesions. A retrospective analysis of 630 cases of neck masses in Turkey revealed that 33.49% were inflammatory, 18.9% were congenital, and 47.6% were neoplastic. However, PTC accounted for only 2% of neoplastic neck masses and 0.9% of all neck masses (6, 7).

While most lateral neck cysts are benign, metastatic lymph nodes from PTC may present as isolated neck cysts. According to investigations, the incidence of occult malignant thyroid tumors in patients with lateral neck cysts is approximately 11%, with an average age of 29 years. Therefore, consideration should be given to the possibility of occult thyroid cancer with cystic lymph node metastasis. It is noteworthy that, in our review of previous cases, reports of ectopic PTC within BCC numbered fewer than 10. Moreover, the thyroid imaging was mostly normal, and the surgical specimens from prophylactic thyroidectomy were mostly negative. PTC was only identified during postoperative pathological examinations of BCC. Cases of BCC with concurrent PTC were reported in less than 5 instances, with typical benign imaging characteristics of BCC. Only two cases displayed mild complexity in imaging features associated with BCC (wall thickening, enhancement, poorly calcified and septated development) (8). In these cases (**Table 1**), the presence of papillary thyroid carcinoma was identified postoperatively through pathological examination of the BCC, prompting further evaluation of the thyroid, and ultimately leading to the discovery of thyroid nodules.

**Table 1:** Summary of partial cases of concurrent PTC and BCC occurrence.

Author	Year	Patient	Medical examination	pathological findings
Balasubramaniam et al. (9)	1992	34(F)	CT: 2.5cm cystic mass in the left neck (thyroid not examined).	Ectopic PTC within a BCC
Hung-Sheng Chi et al. (10)	2007	38(M)	CT: 4.5*5.5cm cystic mass in the right neck (thyroid not examined).	BCC with concurrent PTC
Juri Park et al. (11)	2010	49(M)	CT: 3.2*2.3*4.5 cm cystic mass with focal areas of high-density solid component in the right neck; no thyroid abnormalities identified.	Ectopic PTC within a BCC
Tazegul G et al. (7)	2018	22(F)	MRI: 3.5*2.0cm cystic neck mass; 0.9cm nodule in the right thyroid lobe.	BCC with concurrent PTC
Andy Cooc et al. (8)	2020	49(F)	CT: Large unenhanced cystic lesion in the right neck; heterogeneous cystic thyroid with multiple punctate calcifications.	Bcc with concurrent PTC

For patients over the age of 40, the possibility of lateral neck cysts representing metastatic lymph nodes should always be considered, and efforts should be made to identify the primary tumor before initiating neck treatment (12). Therefore, upon initially receiving this patient, we performed thyroid and cervical lymph node ultrasonography. We identified a solid nodule in the right lobe of the thyroid, measuring approximately 0.4*0.6 cm, with irregular margins and a transverse-to-longitudinal ratio greater than 1 (ACR T1-RADS 5). On neck CT examination, we observed septations, punctate, and arc-shaped calcifications within the neck mass. The solid nodule showed significant enhancement on contrast-enhanced scans. Multiple small lymph nodes were noted in bilateral neck and subclavicular regions. In comparison to previous cases, our success lies in the timely utilization of imaging techniques to examine the patient’s thyroid, identifying thyroid and cervical lymph node lesions, and promptly providing surgical treatment to the patient. To the best of our knowledge, this is the only case providing a detailed description of the occurrence of papillary thyroid carcinoma within a BCC, along with evidence of primary thyroid tumor and lymph node metastasis.

Surgery is the preferred treatment for BCC with concurrent PTC and lymph node metastasis. For BCC occurring in adults, complete excision of the entire fistula and partial thyroidectomy (if the thyroid is involved or to assist in preserving the recurrent laryngeal nerve) appears to be the optimal choice of treatment (13). For children under the age of 8, endoscopic treatment is recommended. In cases where PTC is concurrent, we suggest performing unilateral thyroidectomy with isthmus resection and functional neck lymph node dissection. In this case, the pathological diagnosis from the excisional biopsy of the mass revealed a BCC with concurrent PTC metastasis. During surgery, we performed lymph node dissection in the neck, revealing metastasis in the right central and right neck level III lymph nodes.

## Conclusion

A branchial cleft cyst with concurrent papillary thyroid carcinoma and lymph node metastasis is an extremely rare malignant tumor, with almost no specific clinical and radiological presentations. Particularly for papillary thyroid carcinoma, it is prone to misdiagnosis and missed diagnosis. Therefore, we recommend timely appropriate thyroid examinations before surgery. Sufficient collection of histopathological specimens combined with immunohistochemical results can improve diagnostic accuracy. Surgery is the only effective treatment method. Whether postoperative radioactive iodine therapy should be performed remains controversial, and specific treatment standards await further research.

## Data availability statement

The original contributions presented in the study are included in the article/supplementary material. Further inquiries can be directed to the corresponding authors.

## Ethics statement

Written informed consent was obtained from the individual(s) for the publication of any potentially identifiable images or data included in this article.

## Author contributions

W-TW: Writing – original draft, Investigation, Validation. X-HN: Conceptualization, Software, Writing – original draft. Y-XG: Writing – original draft, Validation, Visualization. RA: Supervision, Writing – original draft. C-LW: Writing – review & editing. JZ: Writing – review & editing.
